# Association between *ACTN3* and acute mountain sickness

**DOI:** 10.1186/s41021-019-0133-8

**Published:** 2019-12-10

**Authors:** Ricardo Muller Bottura, Giscard Humberto Oliveira Lima, Debora Cristina Hipolide, João Bosco Pesquero

**Affiliations:** 1 Department of Psychobiology, UNIFESP, Botucatu Street, 862, First Floor, Vila Clementino, SP, ZIP, São Paulo, 04023062 Brazil; 2 Department of Biophysics, UNIFESP, São Paulo, Brazil

**Keywords:** ACTN3, Acute Mountain sickness, Acclimatization, Altitude, Hypoxia, Hypoxemia, Muscle Fiber types

## Abstract

**Background:**

During the process of acclimatization, when our organism needs to adjust several metabolic processes in the attempt of establishing a better oxygenation, it is normal that individuals present some symptoms that can lead to the disease of the mountain. However, not everyone presents such symptoms and individuals native of high altitudes regions present genetic differences compared to natives of low altitudes which can generate a better acute adaptation. One of these differences is the higher proportion of type I muscle fibers, which may originate from the R577X polymorphism of the *ACTN3* gene. The aim of this study was to compare the response of individuals with different *ACTN3* genotypes at simulated 4500 m altitude on the presence of Acute Mountain Sickness (AMS) symptoms. Twenty-three volunteers (RR = 7, RX = 8, XX = 8) spent 4 hours exposed to a simulated altitude of 4500 m inside a normobaric hypoxia chamber. Lactate and glucose concentrations, SpO_2_, heart rate and the symptoms of AMS were analyzed immediately before entering the chamber and at each hour of exposure. Statistical analysis was performed using IBM SPSS Statistics 21 software.

**Results:**

Our results point to an association between AMS symptoms and the presence of R allele from R577X polymorphism.

**Conclusion:**

We conclude that individuals with at least one R allele of the R577X polymorphism seems to be more susceptible to the effects of hypoxia during the acclimatization process and may develop AMS symptoms.

## Introduction

While we ascend high altitudes, the pressure of oxygen present in the air descends. This phenomenon is known as hypobaric hypoxia and results in decreased pressure of oxygen in arterial blood, which is called hypoxemia [1]. An environment of hypobaric hypoxia, as found at high altitudes, affects several body systems even if exposure to this environment is acute (7 days) [2, 3]. The simple fact of ascending and exposing itself to a certain altitude causes our body to promptly initiate a series of adaptations to compensate for the decrease in the supply of oxygen [1, 4, 5], such as increased heart rate [6], use of glucose as substrate [7, 8] and consequent increase in lactate [9]. At 4500 m, the real amount of oxygen in the air composition is only 12% diluted, which is approximately 60% of sea level oxygen [10]. This lower oxygen pressure causes arterial desaturation (hypoxemia) that is perceived by peripheral chemoreceptors [11] causing increased ventilation and increased sympathetic activation [12], raising heart rate and cardiac output [13]. There are many people who seek high altitudes, whether for work, as miners in Chile and Peru that reach 4500 m [14] or only for sport or entertainment [15]. This high frequency of people at high altitudes between 4500 m and 5000 m brings concern, since 50% of them end up suffering from AMS symptoms [16], which involves headache, fatigue, dizziness, anorexia and nausea. Headache is the most frequent symptom [17], caused mainly by the decrease in arterial oxygen saturation [18–20]. The AMS diagnosis is made by a self-assessment questionnaire called the Lake Louise Score [21]. For the diagnosis it is necessary that the individual is above 2500 m and has the presence of at least two symptoms, one of them being headache [17]. The factors that predispose individuals to AMS symptoms are focus of several studies, including the search for some genetic relation, but without any conclusive results [22]. There are still many questions to be answered about the effects of different altitudes in the acclimatization process, but the literature helps to understand the chronic effects of hypoxemia, especially when analyzing populations living in high altitude regions. The Sherpas, Tibetans [23] and Quechuas [24] have a higher proportion of type I muscle fibers than individuals born in lower regions. These fibers have a higher mitochondrial density than type II fibers and this relationship between the distribution of muscle fiber types and populations born at high altitudes presupposes a genetic predisposition. In addition to the mitochondrial density difference between muscle fiber types, there is a structural difference in the sarcomere of these fibers. The actin filament binds to the Z-line by a protein that is found in two forms in skeletal muscle: α-actinin (ACTN2 and ACTN3), ACTN2 being present in all fiber types, whereas ACTN3 is present only in type II fibers, especially type IIx fibers [25, 26]. However, the *ACTN3* gene undergoes a mutation that results in the exchange of an arginine by a stop codon at position 577 of the protein causing some individuals not to express ACTN3, which is a hereditary deficiency [25]. However, little is known about the effects of acute exposure to hypoxia in relation to genetic differences and therefore it is necessary to investigate in more detail how different genotypes can influence the acclimatization process. Therefore, the aim of this study was to verify the influence of the R577X polymorphism of the *ACTN3* gene on the symptoms of the AMS during the process of acclimatization in healthy individuals exposed 4 hours to simulated high altitude (4500 m).

## Materials and methods

### Participants

This study was carried out in two stages, the first of which was the collection of epithelial cells from the buccal mucosa from 61 volunteers (39 men and 22 women) for the genetic evaluation of the *ACTN3* R577X polymorphism, with twenty-one RR (34.4%), thirty-two RX (52.5%) and eight XX (13.1%). In addition, volunteers responded to the International Physical Activity Questionnaire (IPAQ). The second stage was carried out in the premises of Clube Escola of Universidade Federal de São Paulo (UNIFESP). To participate in the study, volunteers had to be physically active, according to the IPAQ and between 17 and 45 years of age and have no history of cardiovascular diseases. Volunteers who were exposed to altitudes above 2500 m up to 6 months prior to the experiment were excluded from the study. The *ACTN3* distribution of volunteers participating to the second stage was RR = 7, RX = 8 and XX =8.

### Experimental design

**Fig. 1:**
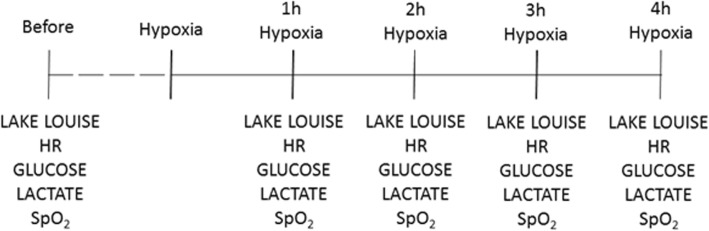
Experimental Design. Participants were evaluated at five different times, the first before entering the camera. Heart Rate (HR), glucose, lactate and peripheral oxygen saturation (SpO_2_) were measured at each moment. Participants presented for the procedure in groups of three, four or five people at a time, randomly among the genotypes

After the first stage of the experiment, individuals returned to the laboratory for the experimental procedure inside the normobaric chamber to 4500 m of altitude. In this situation, volunteers were evaluated in five different moments: (1) immediately before entering the chamber, (2) 1 hour of exposure to hypoxia, (3) 2 hours of exposure to hypoxia, (4) 3 hours of exposure to hypoxia and (5) 4 hours exposure to hypoxia. Blood samples were collected at each time point for evaluation of plasma lactate and glucose concentrations; heart rate (HR) and peripheral oxygen saturation (SpO_2_) were measured at each moment (Fig. 1). All volunteers were evaluated in the morning and received a standard snack after the first analysis before entering the chamber.

### DNA EXTRATION

To determine the genotype of the *ACTN3* R557X gene, epithelial cells from the buccal mucosa were collected using a swab on the inside of the cheek, for approximately 30 s. The samples were then stored in a cooled thermal box to preserve the material at low temperature (4 °C) until it was transported to the laboratory. This collection method was chosen because it is a quick, low-invasive, painless, low-cost procedure and still provides a quality genomic DNA sample. Genomic DNA was extracted using the Chelex 100 resin following the manufacturer’s recommended protocol [27].

### DNA quantification

The quantification of the genomic DNA was performed in a NanoDrop®-ND1000 spectrophotometer. This equipment evaluates the amount of DNA, uses small volumes (1 μL) without the need of dilution and provides parameters to evaluate its purity, regarding the presence of proteins and phenolic compounds [28].

### qPCR GENOTYPING

The SNP (single nucleotide polymorphism) analyzes of the *ACTN3* gene were made by the qPCR allele discrimination method (quantitative PCR) using the TaqMan® SNP genotyping assays (Applied Biosystems, Foster City, CA) and QuantStudio 5 (Applied Biosystems®). The technique allows the analysis of the two variant alleles of a SNP in a particular segment of DNA. For this experimental approach, TaqMan® assays that have already been functionally tested (*ACTN3* rs1815729) were used, because they offer better performance and more affordable cost compared to the customization of the tests [29].

### NORMOBARIC altitude chamber

The Altitude Simulator (CAT - Colorado Altitude Training™/12 CAT-Air Unit) simulates up to 4500 m. This equipment has two air units installed outside the chamber, which performs the gas exchange, increasing nitrogen and reducing O_2_. This gas exchange generates difference in the O_2_ concentration inside the chamber that is visible in a display that shows the simulated altitude in real time, measured by a module that contains an O_2_ cell sensitive to its variations.

### AMS symptoms

In order to diagnose AMS, we used a self-assessment questionnaire called the Lake Louise Score [21, 30], which consists in punctuating from zero to three (0 = absence, 1 = mild, 2 = moderate; 3 = severe) the presence of four symptoms (headache, nausea or lack of appetite, fatigue or weakness, dizziness). For the diagnosis it is necessary that the individual is above 2500 m and has the presence of at least two symptoms (totalizing at least 3 points), one being a headache (with at least 1 point) [17, 30].

### Physiological variables

Peripheral oxygen saturation (SpO_2_) was monitored using a Fingerpulse® finger oximeter, model MD300C202, using a dual light source (a red LED and a red infrared LED) and a photodetector that detects variations in light absorption in the arterial blood, since other tissues such as bones and venous vessels normally absorb a constant amount of light over time. The wavelength of the LED is 660 nm and the infrared LED is 905/880 nm with a maximum optical output power of 4 mW. Heart rate (HR) was measured using the same Fingerpulse® finger oximeter model MD300C202 since the use of this type of appliance at rest is adequate [31]. The glucose and lactate evaluations were performed through blood sample collected from the tips of the fingers. At each collection the fingertips were sanitized with BIOSOMA alcohol swab and later drilled with a BIOLAND Model SB-323 auto lancet. The quantification of these variables was performed through reflectance photometry through ROCHE’s Accutrend Plus Monitor, a portable analyzer for the quantitative determination of glucose, lactate, cholesterol and triglycerides [32].

### Statistics analysis

To analyze the association between the physiological variables and our study variable (presence of AMS symptoms), we used a Generalized Estimating Equations (GEE) with Tweedie distribution, in which the values of each time were condensed into a single variable counting 114 values (23 individuals, 5 times, less 1 lactate time which an error occur with the equipment). Tweedie distribution was used to fit our GEE model using the results of Lake Louise Score as our main variable in which the most of responses was zero, since Tweedie distribution fit both discrete and continuous data [33, 34]. The GEE model with Tweedie distribution was used to investigate if a physiological variable or *ACTN3* polymorphism was associated with an increased chance to develop AMS. In the GEE with the presence of *ACTN3* genotypes and with glucose 3 h and glucose 4 h as covariates, the latter entered only the values of these moments. The level of significance was α ≤ 5%. To perform the tests, the IBM SPSS Statistics 21 software was used.

## Results

### Acute mountain sickness

During the 4 h of exposure to low oxygen pressure mimicking high altitude, three volunteers presented scores related to AMS. One of these volunteers (RX; IMC = 24.7 kg/m2) presented a score 4 between the moments of 2 to 3 h, however, without presence of headache, which eliminates the diagnosis. Of the other two volunteers who presented symptoms (both RRs), one of them (BMI = 24.8 kg/m2) had score 4 at the moment 3 h and score 6 at the moment 4 h, while the other volunteer (BMI = 21.7 kg/m2) presented score 4 at the moment 4 h. In both cases, one of the symptoms reported was headache, which validates the diagnosis of AMS. There were no differences between them and the other volunteers of this study for age, weight, height or BMI (Table 1).

**Table 1:** Characteristics of AMS and no-AMS groups

	AMS (*n* = 2)	No-AMS (*n* = 21)
Age (years)	29.50 ± 0.71	28.81 ± 8.60
Weight (kg)	67.00 ± 14.14	70.52 ± 15.30
Height (m)	1.69 ± 0.09	1.68 ± 0.09
BMI (kg/m^2^)	23.29 ± 2.22	24.78 ± 3.49

Note: characteristics of groups represented by mean ± SD with no statistical differences

**Fig. 2:**
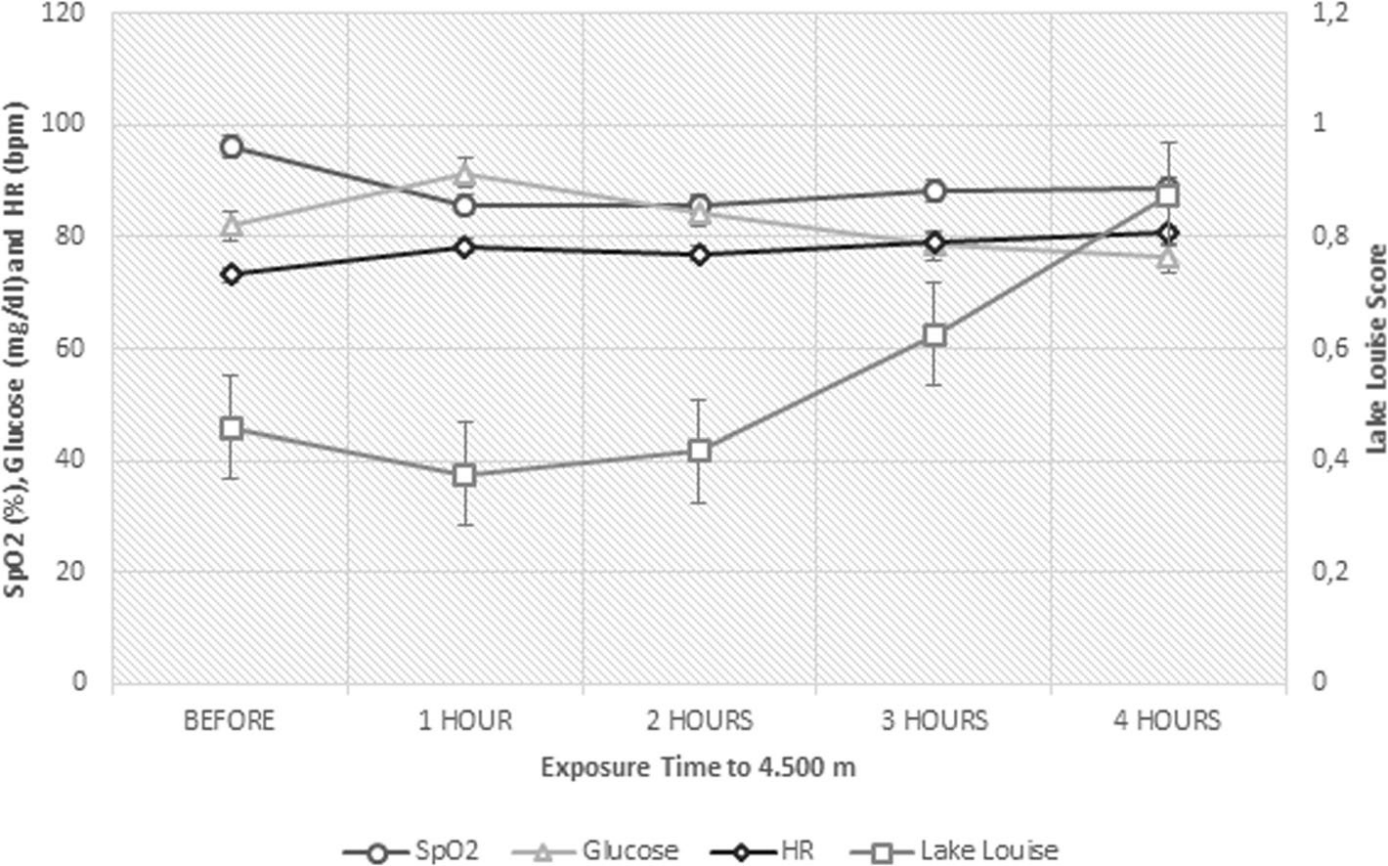
Physiological Variables in relation to *Lake Louise* scores during 4 hours exposure to hypoxia. Relationship between the physiological variables SpO2, glucose and HR with mean Lake Louise scores during the five moments of evaluation. The variable lactate did not enter the graph because it did not show relation to the score or change over time. Values ​​represent the mean of ALL volunteers (*n* = 23) at each time

In order to verify which physiological variables could explain the presence of AMS symptoms, we performed a GEE in which we used the HR, glucose, lactate and SpO_2_ variables analyzed during the whole time of exposure to hypoxia (*N* = 114) and we found only SpO_2_ association [X^2^ (1, N = 114) = 3.58, *p* = 0.05] with the symptoms reported through the Lake Louise questionnaire. This result indicates that there is an inverse relationship between the variables, meaning that the lower the value of SpO_2_, the higher the probability of the volunteer to report a symptom in the questionnaire (β = − 0.04, 95% CI = − 0.09/− 0, *P* = 0.05). Figure 2 shows the relationship between the physiological variables and scores of Lake Louise during all exposure time to hypoxia.

### ACTN3 R577X and AMS

To test our hypothesis of the acclimatization difference of the R577X polymorphism of the *ACTN3* gene, we included in the statistical model the ACTN3 and the glucose data after 3 and 4 h of exposure, since it was in these moments that volunteers presented the symptoms of AMS (*N* = 114). The results are shown in Table 2.

**Table 2:** Result of GEE analysis for Lake Louise, SpO_2_, *ACTN3* and glucose in the moments of 3 and 4 h

Variable	Hypothesis Test		
	X^2^	Df	*P*
HR	0.220	1.0	0.63
SpO_2_	5.560	1.0	0.01*
Lactate	0.530	1.0	0.46
ACTN3	15.604	1.0	< 0.01*
glucose 3 h	0.002	1.0	0.96
glucose 4 h	2.938	1.0	0.08

Note: The association between the physiological variables, the *ACTN3* and Lake Louise scores was verified through a GEE analysis including all values of each volunteer in the five times (before, 1 h, 2 h, 3 h and 4 h), totaling 114 values for each variable. The following variables were included in the statistical model: Heart Rate (HR), Peripheral Oxygen Saturation (SpO_2_), *ACTN3*, lactate, glucose 3 Hours and glucose 4 Hours

*Association with increased symptoms of Lake Louise

The statistical model showed association of AMS symptoms with SpO_2_ [X^2^ (1, *N* = 114) = 5.56, *p* = 0.01] and ACTN3 [X^2^ (1, N = 114) = 15.60, *p* < 0.01]. Considering *ACTN3* genotypes, RX (β = 1.78, 95% CI = 0.62/2.95, *p* < 0.01) and RR (β = 2.08, 95% CI = 1.02/3.13, *p* < 0.01) are associated with Lake Louise, when controlled by the glucose levels after 3 and 4 h. The data expressing these associations are shown in Table 3.

**Table 3:** Result of GEE analysis for Lake Louise, SpO_2_, *ACTN3* polymorphisms and glucose in the moments of 3 and 4 h

Variable	β (95% CI)	*P*
HR	−0.00 (− 0.04, 0.02)	0.63
SpO_2_	−0.04 (− 0.08, − 0.00)	0.01*
Lactate	− 0.19 (− 0.70, 0.32)	0.46
RR	2.08 (1.02, 3.13)	< 0.01*
RX	1.78 (0.62, 2.95)	< 0.01*
XX	0^a^	
glucose 3 h	−0.00 (− 0.02, 0.02)	0.96
glucose 4 h	0.04 (− 0.00, 0.08)	0.08

Note: The association between peripheral oxygen saturation (SpO_2_), *ACTN3* polymorphisms, glucose 3 and 4 Hours, and Lake Louise scores was verified by GEE analysis including all values of each volunteer in the five times (before, 1 h, 2 h, 3 h and 4 h), totalizing 114 values for each variable. The values of genotypes are comparative among them, with XX being the reference value

*CI* Confidence Interval.

*Association between SpO_2_ and RR and RX genotypes with Lake Louise, *p* < 0.05

### Oxygen saturation

In order to investigate which physiological variables could explain the decrease in SpO_2_ over time, we correlated HR, lactate and glucose to verify their behavior against SpO_2_ but did not find any variable that showed significant values.

## Discussion

Several studies have demonstrated the influence of low oxygen pressure at high altitude with AMS, however, in our study we expected that no volunteer would develop AMS. The reason for this assumption is that we have controlled most of the risk factors such as obesity [35], physical effort [36] and low glycemia [37], since our volunteers had a meal just before entering the chamber, did not have any physical activity inside and had normal BMI. Furthermore, our volunteers were exposed only for 4 hours at conditions mimicking 4500 m. Even having controlled all those risk factors, almost 10% of our volunteers had AMS. First we evaluated whether AMS was related to a decreased SpO_2_ since it is the main inducing factor. Interestingly we could demonstrate in our study an association between SpO_2_ with AMS symptoms which corroborates a three-month study in 506 mountaineers of different nationalities in the Alps between 2200 m and 3817 m, that applied the Lake Louise questionnaire and measured SaO_2_ and HR and demonstrated that the main symptom of AMS, headache, is directly related to low SaO_2_, in addition to high perception of effort and low water consumption [18]. Our results reinforce this interaction, since among all the physiological variables analyzed only SpO_2_ was associated with the symptoms reported by the volunteers.

We also analyzed the probability of *ACTN3* being a risk factor to AMS, since both of our volunteers that developed the symptoms had the same genotype (RR). Therefore, the association between the *ACTN3* R577X polymorphism with the presence of AMS symptoms is the main result of our study. Although several genes have been studied in an attempt to find a relationship with the symptoms [22, 38, 39], our study is the first to correlate this condition to *ACTN3* R577X genotypes, showing that individuals with at least one R allele are more susceptible to negative effects of hypoxia during acclimatization. This result, to a certain extent, corroborates previous findings that showed a higher prevalence of X allele in professional mountaineers when compared to normal individuals [40]; higher percentage of type I fibers in populations living in high altitudes such as Quechua of Peru [24] and the Sherpas and Tibetans [23] and in individuals with higher number of capillaries per muscle cross-sectional area [41], which could facilitate the supply of oxygen to the active muscles. Individuals expressing ACTN3 have at least one R allele, and homozygous (RR) individuals have a higher number of type IIx fibers than individuals with the XX genotype, with more type I fibers [42]. In addition, according to Hoppeler and Vogt [43], exposure to hypoxia causes a decrease of about 30% in mitochondrial density without a decrease in capillary density, which would lead to an increase in the supply of oxygen to mitochondria, favoring aerobic metabolism.

The association demonstrated in our study between the symptoms of AMS and *ACTN3* genotypes is related to the decrease of SpO_2_, which is mainly responsible for the presence of AMS symptoms, with headache as the main symptom [18, 19]. This data reinforces our hypothesis that the XX genotype of *ACTN3* may have a faster acclimatization in situations of extreme altitude.

## Conclusion

We conclude that there may be an association between the R allele of the *ACTN3* R577X gene polymorphism and the susceptibility to develop the symptoms of AMS caused by a decreased SpO_2_.

## Limitations of the study

Due to the fact that our study involves genetic variants, the number of analyzed volunteers may not be significant to show a definitive association. Furthermore, in a normobaric chamber, where adverse situations such as cold, wind and physical activity involved in the process of climbing at high altitudes are not present, the effects presented here may have been suppressed. Therefore, based on these restrictions, we suggest that new studies should be performed by other groups in hypobaric hypoxia in order to confirm the findings of this study.

## Data Availability

The datasets used and/or analysed during the current study are available from the corresponding author on reasonable request.

## Abbreviations

ACTN2: Alpha actinin 2
ACTN3: Alpha actinin 3
*ACTN3*: Alpha actinin 3 gene
AMS: Acute Mountain Sickness
BMI: Body Mass Index
GEE: Generalized Estimating Eqs.
HR: Heart Rate
iPAQ: International Physical Activity Questionnaire
SaO_2_: Arterial Oxygen Saturation
SpO_2_: Peripherical Oxygen Saturation
